# 地西他滨在糖皮质激素治疗失败原发免疫性血小板减少症患者中的疗效及影响因素

**DOI:** 10.3760/cma.j.issn.0253-2727.2023.07.008

**Published:** 2023-07

**Authors:** 均蕙 杨, 美娟 薛, 先雷 张, 之琛 韦, 琳琳 邵, 艳 石, 明 侯

**Affiliations:** 山东大学齐鲁医院血液科，济南 250012 Department of Gastroenterology, Qilu Hospital of Shandong University, Jinan 250012, China

**Keywords:** 原发免疫性血小板减少症, 地西他滨, 疗效, 影响因素, Primary Immune Thrombocytopenia, Decitabine, Efficacy, Influencing Factor

## Abstract

**目的:**

评估地西他滨（DAC）在糖皮质激素治疗失败原发免疫性血小板减少症（ITP）中的疗效及影响因素。

**方法:**

纳入2015年11月至2021年6月在山东大学齐鲁医院血液科接受DAC治疗（5 mg·m^−2^·d^−1^×3 d静脉滴注，至少应用3个疗程，每疗程间隔3～4周）的61例糖皮质激素治疗失败ITP患者，对其临床资料进行回顾性分析。

**结果:**

61例患者中，男20例，女41例。中位年龄45（15～81）岁。糖皮质激素依赖43例，无效18例。DAC治疗后，12例（19.67％）患者获得完全反应（CR），16例（26.23％）有效（R），总有效（OR）率为45.90％（28/61）。DAC治疗OR组（28例）与无效组（NR，33例）比较，糖皮质激素的反应性（依赖或无效）、治疗前血小板计数差异具有统计学意义（*χ*^2^＝8.789，*P*＝0.003；*z*＝−2.416，*P*＝0.016）。糖皮质激素依赖组与糖皮质激素无效组比较，DAC治疗2、3疗程后血小板计数较高（*P*＝0.032、0.024）；DAC治疗1、2、3疗程OR率比较，糖皮质激素依赖组均高于糖皮质激素无效组（*P*＝0.042，*P*＝0.012，*P*＝0.029）。糖皮质激素依赖与DAC疗效具有明显相关性（*OR*＝9.213，95％ *CI* 1.937～43.820，*P*＝0.005）。

**结论:**

DAC对于部分糖皮质激素治疗失败的ITP患者具有确切的疗效且不良反应轻微。糖皮质激素依赖、治疗前血小板计数高的患者DAC疗效较好。

## Abstract (en)

**Objective:**

This study aimed to evaluate the efficacy of decitabine（DAC）and identify factors influencing treatment responses in patients with primary immune thrombocytopenia（ITP）who had failed glucocorticoid therapy.

**Methods:**

Clinical data of 61 patients with glucocorticoid-resistant ITP who received DAC therapy（5 mg·m^−2^·d^−1^×3 d via intravenous infusion）for at least three cycles with 3–4-week intervals at the Department of Hematology, Qilu Hospital of Shandong University, from November 2015 to June 2021 were analyzed retrospectively.

**Results:**

The 61 patients comprised 20 males and 41 females, with a median age of 45 years（range: 15–81 years）. Among them, 43 patients were glucocorticoid-dependent（glucocorticoid-dependent group）, while 18 patients were glucocorticoid-resistant（glucocorticoid-resistant group）. Following DAC treatment, 12 patients（19.67％）achieved complete response（CR）, and 16 patients（26.23％）exhibited response（R）, resulting in an overall response（OR）rate of 45.90％（28/61）. Comparison between the OR group（*n*＝28）and the non-response（NR）group（*n*＝33）revealed significant differences in responses to glucocorticoids（dependent or resistant）and platelet counts before treatment（*χ*^2^＝8.789, *P*＝0.003; *z*＝−2.416, *P*＝0.016）. The glucocorticoid-dependent group showed higher platelet counts than the glucocorticoid-resistant group after the second and third cycles of DAC treatment（*P*＝0.032, 0.024）. Moreover, the OR rates after the first, second, and third cycles of DAC treatment in the glucocorticoid-dependent group were all higher than those in the glucocorticoid-resistant group（*P*＝0.042, *P*＝0.012, *P*＝0.029）. A significant correlation was observed between glucocorticoid dependence and responses to DAC treatment（*OR*＝9.213, 95％ *CI* 1.937–43.820, *P*＝0.005）.

**Conclusion:**

DAC demonstrates definitive efficacy with mild adverse effects in a subset of patients with glucocorticoid-resistant primary ITP. Glucocorticoid dependence and higher platelet counts before treatment are associated with a favorable response to DAC therapy.

原发免疫性血小板减少症（ITP）是一种获得性免疫性出血性疾病，以血小板计数减少为主要特点。其一线治疗为糖皮质激素和静脉注射免疫球蛋白（IVIg），二线治疗包括促血小板生成药物、利妥昔单抗、脾切除等[1]。以往研究显示，约80％的患者一线治疗无效或糖皮质激素依赖（减量/停药后复发），此类患者使用二线药物治疗的有效率为60％～70％，最终约1/3的ITP患者对一线、二线治疗无效[2]。既往研究发现，小剂量地西他滨（decitabine, DAC）能够促进巨核细胞的成熟以及血小板生成，成为ITP治疗的新曙光[3]，在临床上应用逐渐广泛。因此我们进行了一项回顾性研究，分析DAC对糖皮质激素治疗失败ITP患者的疗效及相关影响因素，为DAC的合理应用提供循证证据。

## 病例与方法

一、一般资料

本研究纳入2015年11月至2021年6月山东大学齐鲁医院血液科收治的糖皮质激素治疗失败ITP患者，所有患者符合《成人原发免疫性血小板减少症诊断与治疗中国指南（2020年版）》[1]诊断标准。男20例，女41例，中位年龄45（15～81）岁；糖皮质激素依赖43例，无效18例。DAC二线治疗25例，三线及以上治疗36例。新诊断ITP 15例，持续性ITP 17例，慢性ITP 29例。

二、治疗方案

DAC 5 mg·m^−2^·d^−1^静脉滴注，连续3 d为1个疗程，每例患者至少应用3个疗程，每疗程间隔3～4周，有效者再巩固3～6个疗程。

三、随访

采用门诊、住院、电话等方式随访，随访记录至少3个月的相关信息，中位随访4（3～73）个月，随访终止日期为2021年12月31日。

四、疗效评价标准

根据《成人原发免疫性血小板减少症诊断与治疗中国指南（2020年版）》[1]来进行疗效判断，分为完全反应（CR）、有效（R）和无效（NR），获得CR和R患者在全部患者中的占比（％）为总有效（OR）率。

五、统计学处理

使用SPSS24.0、Graphpad Prism9.4.0进行数据分析与绘图，连续变量采用M-W秩和检验，以“中位数（范围）”表示；分类变量采用卡方检验和Fisher精确检验，以“例数”和“百分比”表示；相关性比较采用二元Logistic回归，*P*<0.05认为差异具有统计学意义。

## 结果

一、疗效与安全性

61例患者中，分别有12例（19.67％）、获得CR，16例（26.23％）获得CR、R疗效，OR率为45.90％（28/61）。中位随访时间4（3～73）个月，中位起效时间28（7～56）d，中位疗效维持时间为5.5（1～72）个月。出现轻微脱发1例，未发生血液系统损伤、恶心、呕吐等不良反应。

二、DAC治疗OR组与NR组临床指标比较

OR与NR组比较，糖皮质激素反应性、治疗前血小板计数差异具有统计学意义（*χ*^2^＝8.789，*P*＝0.003；*z*＝−2.416，*P*＝0.016），详见表1。

**表1:** 地西他滨（DAC）治疗有效（OR）与无效（NR）糖皮质激素治疗失败ITP患者临床指标比较

项目	OR组（28例）	NR组（33例）	*χ^2^/z*值	*P*值
性别（男/女）	10/18	10/23	0.201	0.654
年龄［岁，*M*（范围）］	40（17~81）	52（15~74）	−0.652	0.515
病程［月，*M*（范围）］	7.5（1~240）	24（0.5~360）	−1.289	0.197
疾病分期［例（％）］			3.947	0.139
新诊断ITP	7（25.00）	8（24.24）		
持续性ITP	11（39.29）	6（18.18）		
慢性ITP	10（35.71）	19（57.58）		
糖皮质激素治疗反应［例（％）］			8.789	0.003
依赖	25（89.29）	18（54.55）		
无效	3（10.71）	15（45.45）		
治疗前PLT［×10^9^/L，*M*（范围）］	11（1~42）	5（0~37）	−2.416	0.016
骨髓巨核细胞数［个，*M*（范围）］	77.5（2~500）	175（0~1 200）	−1.699	0.089
血小板抗体［例（％）］			0.421	0.138
阴性	10（66.67）	7（43.75）		
Ⅰb阳性	2（13.33）	0（0.00）		
Ⅱb/Ⅲa阳性	1（6.67）	3（18.75）		
Ⅰb、Ⅱb/Ⅲa双阳性	2（13.33）	6（37.50）		

**注** ITP：原发免疫性血小板减少症

三、DAC二线治疗组与≥三线治疗组临床疗效指标比较

DAC二线治疗组2疗程血小板计数、OR率均高于≥三线治疗组（*z*＝−1.989，*P*＝0.047；*χ*^2^＝3.949，*P*＝0.047）。详见表2。

**表2:** 以DAC二线治疗组与≥三线治疗组疗效指标比较

项目	二线治疗（25例）	≥三线治疗（36例）	*χ^2^/z*值	*P*值
性别（男/女）	9/16	11/25	0.198	0.656
年龄［岁，*M*（范围）］	50（17~74）	45（15~81）	−0.609	0.543
基线PLT［×10^9^/L，*M*（范围）］	9（0~42）	5.5（1~30）	−0.779	0.436
1疗程PLT［×10^9^/L，*M*（范围）］	31（1~235）	15（1~177）	−0.345	0.730
1疗程总有效率［例（％）］	10（40.00）	8（20.22）	2.242	0.134
2疗程PLT［×10^9^/L，*M*（范围）］	32（1~150）	10.5（1~195）	−1.989	0.047
2疗程总有效率［例（％）］	14（56.00）	11（30.56）	3.949	0.047
3疗程PLT［×10^9^/L，*M*（范围）］	27（1~163）	8.5（1~150）	−1.615	0.106
3疗程总有效率［例（％）］	9（36.00）	10（27.78）	0.465	0.495

四、糖皮质激素依赖组与糖皮质激素无效组临床疗效指标比较

糖皮质激素依赖组与糖皮质激素无效组比较，基线和DAC治疗1疗程血小板计数差异无统计学意义（*P*＝0.303，*P*＝0.203），DAC治疗2、3疗程中位血小板计数较高（*P*＝0.032，*P*＝0.024）。DAC治疗1、2、3疗程总有效率比较，糖皮质激素依赖组均高于糖皮质激素无效组（*P*＝0.042，*P*＝0.012，*P*＝0.029）。详见表3。

**表3:** 糖皮质激素依赖组与糖皮质激素无效组地西他滨（DAC）临床疗效指标比较

指标	糖皮质激素依赖（43例）	糖皮质激素无效（18例）	*χ^2^/z*值	*P*值
性别［例（男/女）］	14/29	6/12	0.003	0.953
年龄［岁，*M*（范围）］	39（15~81）	53（19~74）	−1.171	0.242
病程［月，*M*（范围）］	15（0.67~360）	7（0.5~240）	−1.187	0.235
基线PLT［×10^9^/L，*M*（范围）］	9（0~42）	5.3（1~27）	−1.030	0.303
1疗程PLT［×10^9^/L，*M*（范围）］	22（1~235）	12.5（2~89）	−1.274	0.203
1疗程总有效率［例（％）］	16（37.21）	2（11.11）	4.155	0.042
2疗程PLT［×10^9^/L，*M*（范围）］	30（1~195）	7.5（1~136）	−2.145	0.032
2疗程总有效率［例（％）］	22（51.16）	3（16.67）	6.243	0.012
3疗程PLT［×10^9^/L，*M*（范围）］	27（2~163）	8（1~80）	−1.995	0.024
3疗程总有效率［例（％）］	17（39.54）	2（11.11）	4.780	0.029

五、DAC疗效影响因素的相关性分析

将DAC疗效与性别、年龄、病程、疾病分期、激素反应性、DAC是否为二线治疗、治疗前血小板计数等影响因素进行相关性分析，结果显示糖皮质激素依赖与DAC有效具有明显相关性（*OR*＝9.213，95％ *CI* 1.937～43.820，*P*＝0.005）。

## 讨论

ITP是临床常见的出血性疾病，成人年发病率为（2～10）/10万，60岁以上老年人为高发群体，育龄期女性略高于同年龄组男性[1]。主要发病机制是患者对自身抗原免疫耐受性丧失，致使体液免疫和细胞免疫活化异常，共同介导血小板破坏增加及血小板产生减少[4]。早期研究表明，在巨核细胞分化过程中起调控作用的糖蛋白Ⅵ（GPⅥ）表达受DNA甲基化的调节[5]，近年来DNA甲基化在ITP中多种机制被发现，异常的DNA甲基化调控相关基因的表达，导致巨核细胞分化成熟障碍和血小板破坏增多，最终造成血小板的减少[6]–[8]。

DAC作为一种经典的去甲基化药物，在临床上应用广泛，低剂量DAC可通过促进巨核细胞成熟分化、促进血小板释放，以提升血小板数量[9]–[11]。研究发现低剂量DAC通过上调CD8^+^ T细胞上PD-1的表达和恢复PD-1甲基化来抑制CTL的细胞毒性、促进Treg细胞的生成和分化，这是低剂量DAC在ITP获得持续反应的一个可能机制[12]–[13]；低剂量DAC还被证实可通过调节髓源性抑制细胞的代谢引起ITP的持续缓解[14]。

目前DAC属于治疗ITP的三线药物，因此多用于难治/复发性患者，近年研究证实了低剂量DAC对于难治性ITP的有效性与安全性，OR率约为50％，CR率约为20％，不良反应轻微[15]–[16]。在本研究中61例糖皮质激素治疗失败的ITP患者采用DAC治疗，OR率为45.90％，CR率为19.67％，中位起效时间28（7～56）d，不良反应轻微，OR率低于既往研究结果，而CR率、中位起效时间、中位疗效维持时间与既往研究结果相近。在本研究中，DAC二线治疗组（25例）、≥三线治疗组（36例）的OR率分别为60.00％（15/25）、36.11％（13/36），差异无统计学意义。目前治疗ITP二线药物中，促血小板生成药物1～2周起效，有效率60％以上[1]，但价格较高，停药后疗效多不能维持，需长时间用药；利妥昔单抗初始OR率为57％，5年OR率为21％，但起效时间长（6～8周），且发生感染的风险增加[17]。在本研究中，DAC二线治疗组的OR率达60％，1疗程中位血小板计数达31×10^9^/L，不良反应轻微，从用药成本、疗效及安全性综合考虑，DAC二线治疗ITP似乎存在一定的优势，但本研究属于回顾性研究，样本量小，有待于进一步开展前瞻性、多中心系列对照研究进一步明确DAC在ITP二线治疗中的作用。

目前对DAC治疗ITP疗效的预测研究尚少，尤其对于一线糖皮质激素治疗失败的情况下，在选择DAC治疗时尤为被动。一项研究[18]评估了糖皮质激素抵抗型ITP患者血浆可溶性细胞间黏附分子1（ICAM-1）水平与低剂量DAC疗效的相关性，结果提示ITP患者血浆ICAM-1水平对低剂量DAC疗效具有潜在预测价值。在本研究中，糖皮质激素依赖、用药种类少及治疗前血小板计数高的患者，DAC疗效更好；相关性分析中发现，糖皮质激素与DAC有效具有明显的相关性，提示糖皮质激素的反应性对DAC疗效具有一定的预测价值，其具体机制尚需进一步探究。

骨髓中巨核细胞是产生血小板的来源，本研究收集到43例患者的骨髓细胞学结果，发现DAC治疗有效的患者巨核细胞中位数低于DAC无效者，但是两者比较差异无统计学意义。传统认为经典的ITP发病机制是自身抗体介导的血小板减少，但仅60％左右的ITP患者可检测到抗血小板相关抗体[19]。在本研究中收集到31例患者血小板抗体情况，发现血小板抗体差异与DAC疗效之间无统计学意义。另外，性别、年龄、疾病分期、病程与DAC的疗效亦无明显相关性。

综上所述，本研究结果显示，DAC对部分既往糖皮质激素治疗失败的ITP患者具有确切的疗效且不良反应轻微，糖皮质激素依赖、治疗前血小板计数高的患者，DAC疗效更好。本研究为回顾性研究，样本数量不大，上述结论尚需进一步验证。
